# Modeling the temporal prevalence peak drift of chronic diseases

**DOI:** 10.1186/s12874-025-02517-1

**Published:** 2025-03-07

**Authors:** Jürgen Rodenkirchen, Annika Hoyer, Ralph Brinks

**Affiliations:** 1 Biostatistics and Medical Biometry, Medical School OWL, Bielefeld University, Universitätsstr. 25, Bielefeld, 33615 Germany; 2 Chair for Medical Biometry and Epidemiology, Faculty of Health/School of Medicine, Witten/Herdecke University, Alfred-Herrhausen-Str. 50, Witten, 58455 Germany

**Keywords:** Illness-death model, Incidence, Prevalence, Mortality, Lexis plane, Logistic function, Transport equation, Ricatti equation, Implicit function theorem

## Abstract

**Background:**

Chronic diseases, such as type 2 diabetes, are responsible for a substantial proportion of global deaths and are marked by an increasing number of people that suffer from them. Our objective is to systematically investigate the analytical determination of the drift in prevalence peaks over calendar-time and age, with an emphasis on understanding the intrinsic attributes of temporal displacement. This aims to enhance the understanding of disease dynamics that may contribute to refining medical strategies and to plan future healthcare activities.

**Methods:**

We present two distinct yet complementary approaches for identifying and estimating drifts in prevalence peaks.

First, assuming incidence and mortality rates are known, we employ a partial differential equation that relates prevalence, incidence, and mortality. From this, we derive an ordinary differential equation to mathematically describe the prevalence peak drift.

Second, assuming prevalence data (rather than incidence and mortality data) are available, we establish a logistic function approach to estimate the prevalence peak drift. We applied this method to data on the prevalence of type 2 diabetes in Germany.

**Results:**

The first approach provides an exact mathematical prescription of the trajectory of the prevalence peak drift over time and age, assuming incidence and mortality rates are known. In contrast, the second approach, a practical application based on available prevalence data, demonstrates the prevalence peak dynamics in a real-world scenario. The theoretical model, together with the practical application, effectively substantiates the understanding of prevalence peak dynamics in two different scenarios.

**Conclusion:**

Our study shows the theoretical derivation and determination of prevalence peak drifts. Our findings underpin the dynamic nature of chronic disease prevalence, highlighting the importance of considering the related age-dependent trends for planning future healthcare activities.

**Supplementary Information:**

The online version contains supplementary material available at 10.1186/s12874-025-02517-1.

## Background

In 2015, 71% of the 56 million global deaths were attributable to chronic diseases with an upward trend in the past decades [1]. Chronic diseases are characterized by irreversibility of the condition, i.e. the missing opportunity of remission from the diseased state back to the healthy state. Worldwide, diabetes mellitus is one of the most frequent chronic diseases, and thus, is a disease with high public health relevance [2–5]. Currently, type 2 diabetes prevalence is estimated to be $7.4\%$ among men and $7.0\%$ among women in Germany aged 40 years or older [4, 6]. Besides severe late complications, diabetes is associated with an increased mortality and with healthcare costs 1.5 to 4.4 times higher than those for people without diabetes [2].

Considerations for reducing health care costs of chronic diseases require an understanding of the prevalence, which accounts for both calendar-time and age-dependent variations in disease occurrence. Future trends in age-specific prevalence peaks, which refer to the specific ages where the prevalence is highest within a population at a given point in time, must be carefully analyzed to anticipate shifts in healthcare needs and resource allocation. The complex nature of the prevalence peak drift underscores the need to uncover and estimate its dynamic hidden in the data. A thorough methodological investigation of this peak drift in relation to both calendar-time and age is, to the best of our knowledge, currently missing in the literature. This work aims to address this gap by providing a comprehensive analysis of the peak drift through two distinct yet complementary methods: a partial differential approach, suitable when incidence and mortality data are available, and a logistic function approach, which is appropriate if prevalence data are available.

**Fig. 1:**
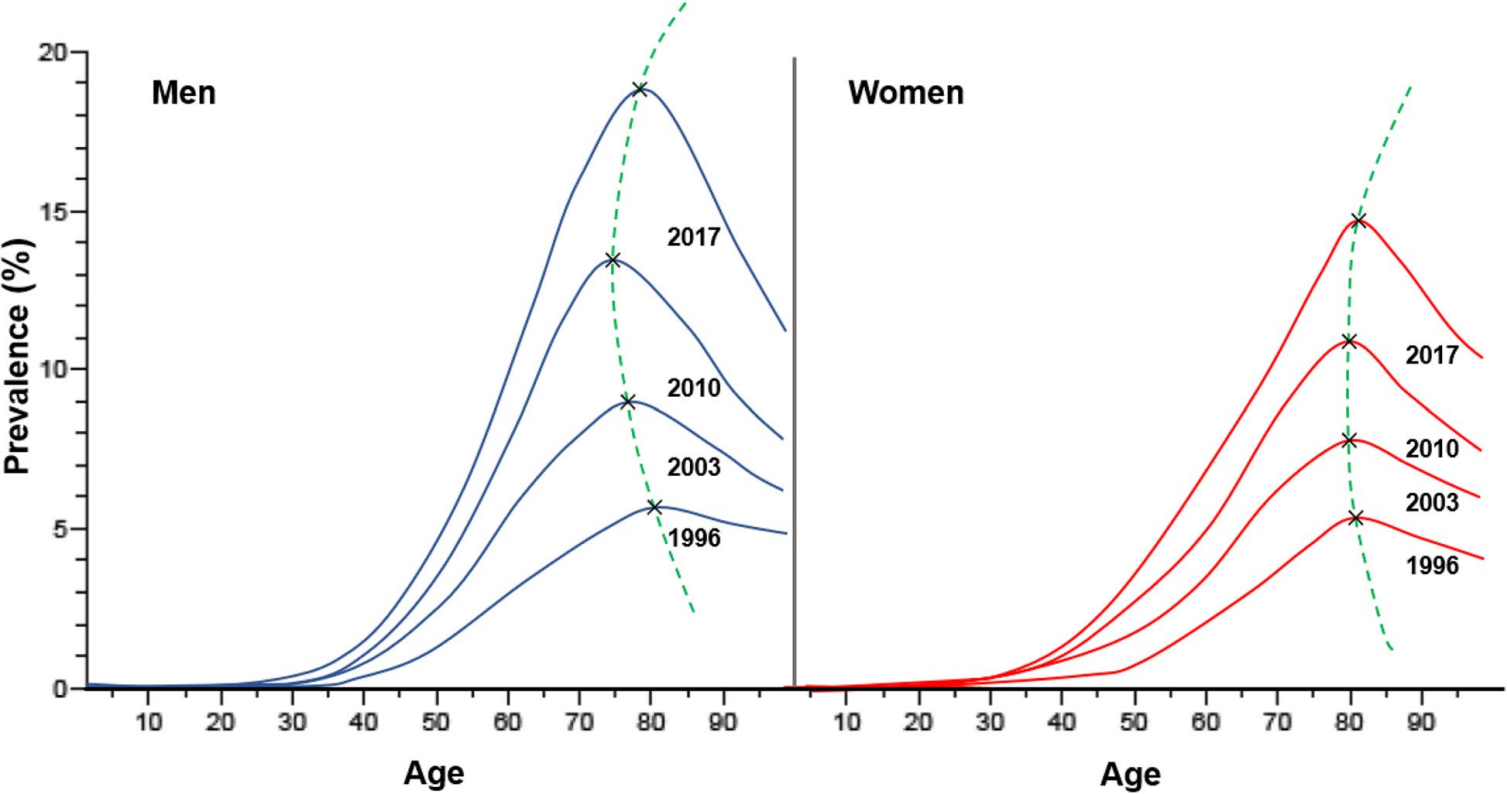
Age-specific prevalence of type 2 diabetes in Denmark as of 1 January 1996, 2003, 2010, 2017. Blue curves (left) are men, red curves (right) women; drift of peak prevalence illustrated by dashed green lines across changing age and calendar-time, highlighted by black crosses

As a motivating example, we focus on the prevalence of type 2 diabetes in Denmark (Fig. 1, adapted from [7], p. 4) which changes over calendar-time and age. The dashed green line, intersecting the black crosses denoting peaks, highlights the shift of prevalence peaks when projected and interpreted in the three dimensional (*t*, *a*, *p*(*t*, *a*)) space. Here, *t* denotes calendar-time, *a* denotes age, and *p*(*t*, *a*) represents the prevalence at calendar-time *t* and age *a*. In the depicted graph, a distinct pattern emerges for the male population. Specifically, the analysis reveals that the maximum of prevalence was attained by men in the year 1996, registering at approximately 5% within the demographic bracket of 80 years. However, as of 2017, this peak has surged notably to around 18%. Notably, the age cohort most vulnerable to this phenomenon remains centered around 80 years. Projections stemming from the extrapolation of the trajectory of these peaks, as delineated by the dashed green line, augur the continuation of the upward trajectory in prevalence peaks during the forthcoming years. Furthermore, this trajectory elucidates a concurrent migration of the age cohort exhibiting these peaks, gradually extending into higher ages within the male demographic. This temporal progression reveals that the age at which prevalence peaks undergoes shifts over time which may have important implications for planning healthcare strategies. To estimate the prevalence peak drift, we explore two distinct yet complementary approaches: a general mathematical approach applying a partial differential equation (cf. Differential equation approach section), which is suitable when incidence and mortality data are available and an application-oriented technique that uses logistic functions (cf. Logistic function approach section), which is applicable when prevalence data are available.

Our starting point and general framework for modeling chronic diseases is based on the illness-death model (IDM), a multistate model that categorizes individuals into three states: Non-diseased, Diseased, and Dead [8, 9]. This model has been around since at least the 1950s [10] and has proven to be an essential tool for analyzing transitions between health states in chronic disease epidemiology. Multistate models, in general, have a long-standing role in epidemiology. For instance, they have been employed in infectious disease research for nearly ninety years [11] and in chronic disease studies for over half a century [10]. Murray and Lopez extended the IDM by introducing a system of ordinary differential equations (ODEs) to describe health state transitions [12]. These ODEs formed the core of the influential *Global Burden of Disease Study*, which has garnered much attention over the past decade [13, 14]. In their work, Murray and Lopez handled disease states in a manner analogous to the Kermack-McKendrick model, now better known as the SIR model in the context of infectious disease epidemiology. Recently, Brinks and Hoyer [15] have shown that the prevalence of chronic diseases can be expressed in terms of the transition rates within the IDM via a scalar partial differential equation (PDE), which serves as the foundation for our current research.

For given mortality and incidence rates, the prevalence drift can be derived at least theoretically from the PDE governing the IDM, since the PDE on the trace (as a 1-dimensional subset of the Lexis plane) becomes a solvable ordinary differential equation (ODE), namely a solvable *Riccati differential equation*. The solution of the Riccati Equation allows us to determine an ODE for the trace using the *Implicit Function Theorem*. However, the solvability of this ODE (and thus the analytical computability of the trace) depends on its structure, and in some cases, it may be impossible to solve due to its sheer complexity. This theoretical approach will be investigated first.

However, in practical applications where only aggregated data on prevalence is available and with limited access to incidence and mortality rates, the second approach provides an alternative. This method uses logistic functions and polynomial data approximation techniques. Therefore, we show how fundamental analytical methods can be used to calculate the trace of the prevalence drift using an example on type 2 diabetes in 70 million German people, where the visual inspection of the aggregated data already indicates the presence of a drift. Furthermore, it should be noted that incidence and mortality rates can successfully be modeled or estimated [16–18], which would allow for additional insights and potential integration with the theoretical differential equation approach.

The article is organized as follows: First, we introduce the differential equation approach and the related trace equation based on given mortality and incidence rates. Subsequently, we demonstrate the existence of a logistic function solution consistent with a given aggregated data set on type 2 diabetes, thereby supporting our theoretical model. Finally, we summarize and discuss our main findings.

The data and R code used for the calculations and simulations presented in this article, along with a how-to guide, are available in the supplementary materials.

## Methods

### Differential equation approach

#### Illness-death model

**Fig. 2:**
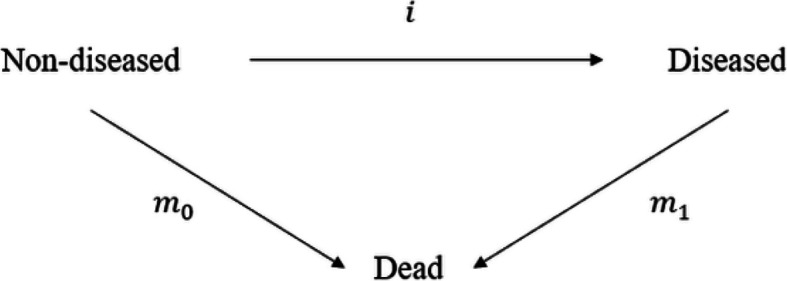
Illness-Death model of a chronic disease. The incidence rate *i*, the mortality rate of non-diseased $m_0$ and the mortality rate of the diseased $m_1$ describe the transitions between the states

Our considerations are based on the IDM shown in Fig. 2. The IDM consists of the three states Non-diseased, Diseased and Dead. The transition rates between these states are given by the incidence rate $i=i(t,a)$, the mortality rate of the non-diseased $m_0 = m_0(t, a)$ and the mortality rate of the diseased $m_1 = m_1(t, a)$. All of them depend on calendar-time *t* and age *a*. Furthermore, we denote the excess mortality rate by

$$\begin{aligned} \Delta m := m_1 - m_0. \end{aligned}$$

The IDM is fully governed by the following PDE that describes the temporal change of the prevalence depending on the incidence and mortality rates [15],

1 $$\begin{aligned} \left( \frac{\partial }{\partial t} + \frac{\partial }{\partial a} \right) p = (1-p)(i - p\Delta m), \end{aligned}$$

where $p=p(t,a)$ denotes the prevalence of the chronic disease for individuals aged *a* at time *t*.

#### The trace equation

We assume that in each calendar year $t_k$ within the observation period $T = [t_0, t_e]$ there is a unique age $a_k = a(t_k)$ at which the prevalence reaches a relative maximum. To derive a PDE-based model, we extend this assumption by proposing that at each point in the continuum $t \in T$, there exists a unique age $a = a(t)$ at which the prevalence reaches its maximum. We are then interested in the continuous function $a=a(t)$, which describes the trajectory of prevalence peaks in the Lexis plane. Assuming the prevalence is a differentiable function $p = p(t,a)$, the necessary condition for a relative maximum is provided by Eq. (3).

Furthermore, we assume that the prevalence can be computed using the partial differential Eq. (1), given that incidence rate $i = i(t,a)$ and mortality rates $m_k = m(t,a), \, k = 0,1$ are known. From Eq. (3), we can directly derive Eq. (4) by substituting (3) into (1). Together, (3) and (4) form a system of differential equations, which uniquely determines the trajectory of prevalence peaks in the Lexis plane, if initial conditions for the age *a* at initial time $t = t_0$ are given.

#### Preliminaries and notations

**Fig. 3:**
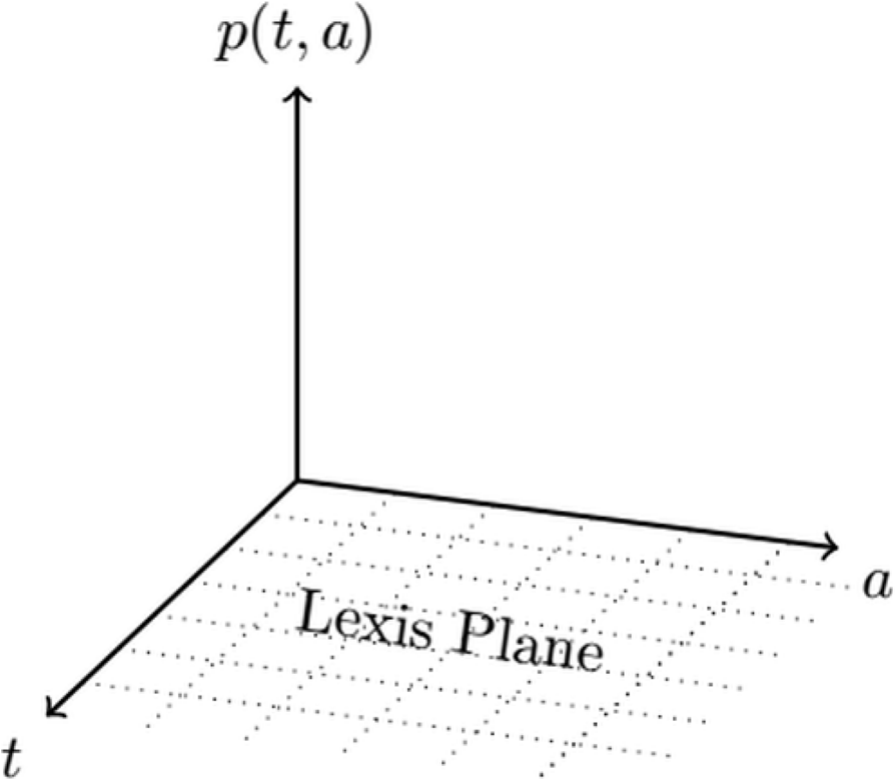
Lexis Plane which encompasses all points in the (*t*, *a*) - plane, where *t* represents calendar-time and *a* denotes age

Figure 3 shows the coordinate setting $(t,a,p(t,a))^T$ with the Lexis plane consisting of the two axis *t* and *a*. The Lexis diagram is a graphical representation used in demography to depict the relationship between calendar-time and age. By “walking through” the Lexis diagram, we can track the progression of individuals or cohorts in terms of time and age, allowing us to analyze patterns, trends, and demographic processes. It therefore provides insights into ageing, generational shifts, and demographic developments.

Let $p=p(t,a)$ be a sufficiently smooth solution of the PDE (1). To determine the trace of the prevalence drift in the Lexis plane, we determine for each calendar-time point $t=\bar{t}$ an age $a=a(\bar{t})$ at which the derivative of *p* with respect to *a* vanishes. This requirement is a sufficient condition for the determination of the trace since we can infer from practical considerations that the scalar function

$$\begin{aligned} p(\bar{t},a):\mathbb {R}^{\ge 0} \rightarrow [0,1] \end{aligned}$$

must possess a local maximum for each fixed $t=\bar{t}$. With this in mind, we define

##### Definition 1

Let $J_{CT}$ and $J_{A}$ be the calendar-time and age interval, respectively. A subset of the **solution set** $\mathbb {L} = \left\{ (t,a,p(t,a))~ | ~ t\in J_{CT} \subset \mathbb {R}^{\ge 0} , a\in J_A \subset \mathbb {R}^{\ge 0} \right\}$ with the property that the directional derivative of *p* in the direction $\vec {a}=(0,1)^T$ of the *a* axis vanishes, is called **implicit prevalence drift** or **implicit drift** for short. Additionally, the domain of definition of the implicit drift in the Lexis plane,

2 $$\begin{aligned} \mathbb {T} = \left\{ (t, a(t)) \ | \ t\in J_{CT}, \ \frac{\partial }{\partial a} p(t,a)_{|a=a(t)}=0 \right\} , \end{aligned}$$

is denoted the **trace of the implicit drift** or **trace** for short. The function $a:J_{CT} \rightarrow \mathbb {R}$ for which $\frac{\partial }{\partial a}p(t,a)_{|a=a(t)}=0$ for each fixed $t\in J_{CT}$ is called the **trace function** of the prevalence peak drift.

##### Remark 1

Given the nature of the data, it is reasonable to assume that the approximating prevalence function $p=p(t,a)$ at each calendar-time point *t* exhibits a unique relative maximum with respect to age *a*, and no relative minimum. Therefore, in the context of the current problem, the condition $\frac{\partial }{\partial a}p(t,a)_{|a=a(t)}=0$ can be regarded as both a necessary and sufficient criterion for identifying the desired relative maximum.

##### Remark 2

The prevalence $p$, which corresponds to the solution of the partial differential Eq. (1), is not explicitly known, as the PDE generally cannot be solved analytically. Nevertheless, we will show that a mathematically complete description of the trajectory of p can still be obtained in the form of an ordinary differential equation, without requiring the explicit solution $p$. We refer to this as an “implicite” solution, as the prevalence p is implicitly embedded within the information provided by the PDE. This terminology is mathematically justified, as the derivation of the ODE relies on the application of the “implicite function theorem”.

##### Conditional ODE for the trace

We are mainly interested in the trace function rather than in the prevalence peak function as it contains the information of interest on the future prevalence trends. From (1) and (2) it is clear that the trace function $a=a(t)$ (if it exists) must obey and is completely described by the following system of ODEs:

3 $$\begin{aligned} \frac{\partial }{\partial a}p(t,a) & = 0 \end{aligned}$$

4 $$\begin{aligned} \frac{\partial }{\partial t}p(t,a) & = (1-p)(i - p\Delta m ) . \end{aligned}$$

Equation (4) is an ODE in the variable *t* because here *a* is not the ordinary age variable but denotes the trace $a=a(t)$, i.e. the age as function of the calendar-time *t*. Especially, (4) is a Riccati differential equation with special solution $p(t,a(t))\equiv 1$. As such, it is known to be analytically solvable in that it can be transformed into a linear ODE by a suitable substitution (cf. Additional file 1: Appendix A.pdf; *The Riccati Equation*). Now, let

5 $$\begin{aligned} D=D(t,a(t)) \end{aligned}$$

be a solution of (4). Then, from Eq. (3) we conclude

6 $$\begin{aligned} F(t,a(t)):= \frac{\partial }{\partial a}D(t,a)_{\mid a=a(t)} = 0 . \end{aligned}$$

Finally, under some regularity assumptions at *F*, Eq. (6) can be solved by an application of the *Implicit Function Theorem* (cf. e.g., [19], p. 42 - 48) leading to the conditional ODE for the trace $a=a(t)$:

7 $$\begin{aligned} \left. \begin{array}{l} a'(t) = - \frac{\partial }{\partial t}F(t, a(t)) \cdot \left[ \frac{\partial }{\partial a}F(t, a(t)) \right] ^{-1} \\ F(t,a(t))= \frac{\partial }{\partial a}D(t,a) \end{array}\right\} \end{aligned}$$

From Eq. (7) we may conclude that in order for the trace to exist it is sufficient that $\frac{\partial }{\partial a}F(t,a)\ne 0$ and that the right-hand side of (7) is continuous on the Lexis plane. If the trace exists, it is unique in the sense that exactly one plausible solution among possibly several exists (cf. The calculation of the trace section).

Equation (7) is in general a nonlinear ordinary differential equation, thus it cannot be solved for specific applications. Nevertheless, there is always the possibility of numerical approximation, which should be sometimes sufficient.

### The relationship between mortality and incidence rates

For a clearer distinction of the trace $a=a(t)$ from the ordinary age variable *a*, we will denote the trace with $\hat{a} = \hat{a}(t)$ in the following. We differentiate (1) with respect to *a* and make use of the fact that the solution’s derivative $\frac{\partial }{\partial a}p(t,a)$ vanishes on the trace $\hat{a} = \hat{a}(t)$, i.e

8 $$\begin{aligned} \frac{\partial }{\partial a}p(t,\hat{a}) = \frac{\partial }{\partial a}p(t,\hat{a})_{\mid a=\hat{a}}=0, \end{aligned}$$

which leads us to

9 $$\begin{aligned} \frac{\partial }{\partial a}i\left( t, \hat{a}\right) = p\left( t, \hat{a}\right) \cdot \frac{\partial }{\partial a}\Delta m\left( t, \hat{a}\right) + \frac{1}{1- p(t, \hat{a})} \cdot \frac{\partial }{\partial a} \left( \frac{\partial }{\partial t} + \frac{\partial }{\partial a} \right) p\left( t, \hat{a}\right) . \end{aligned}$$

In cases where the second summand of (9),

10 $$\begin{aligned} \text {Pert}\left( t,\hat{a}\right) := \frac{1}{1- p(t,\hat{a})} \cdot \frac{\partial }{\partial a} \left( \frac{\partial }{\partial t} + \frac{\partial }{\partial a} \right) p\left( t, \hat{a}\right) , \end{aligned}$$

turns out to be small, the following relationship holds

11 $$\begin{aligned} \frac{\partial }{\partial a}i\left( t, \hat{a}(t)\right) \approx p\left( t, \hat{a}\right) \cdot \frac{\partial }{\partial a}\Delta m\left( t, \hat{a}(t)\right) \end{aligned}$$

approximately with proportionality factor $p\left( t, \hat{a}(t)\right)$. Therefore, we may refer to (10) as *perturbation term* because it can be considered as a measure of the perturbation of the linear relationship (11) between the incidence and excess mortality. In the special application of The correspondence between mortality- and incidence rates section, this term actually turns out to be negligibly small. Thus, the change in the incidence rate with age in the case of The correspondence between mortality- and incidence rates section is a linear function of the change in the excess mortality with slope in the order of magnitude of the prevalence of the chronic disease.

## Logistic function approach

The logistic function approach aims to determine the trace of the prevalence drift based on aggregated prevalence data only. Even if the case incidence and mortality rates were known, these measures would still be complex scalar functions dependent on both *t* and *a* [16], for which the nonlinear ordinary differential equation (ODE) (7) does not have an analytical solution. Nevertheless numerical methods are always available and could be applied. But this would require deeper investigation of numerical methods for nonlinear PDEs which is beyond the scope of this article.

For the logistic function approach, we first approximate the data by fitting a polynomial function in terms of *t* and *a*. This allows for a sufficiently exact mathematical representation of the data (Application: type 2 diabetes in Germany section). Secondly, the data is transformed through the application of the logistic function, mapping it from the set of real numbers $\mathbb {R}$ to the interval [0, 1]. This transformation provides a potential solution, denoted as $p=p(t,a)$, to the problem under consideration (On the existence of logistic function solutions section, Lemma 1). Furthermore, the logistic function approach is capable of obtaining an analytical solution to the problem (The calculation of the trace section). As a by-product, it provides evidence for the existence of a prevalence drift.

### On the existence of logistic function solutions

We approximate a given prevalence data set $\left\{ P(t_k, a_k)~|~ k=1, \dots n \right\}$ by a polynomial $f=f(t,a)$. With this polynomial, we then define the function *p* by applying the logistic function to the polynomial *f*,

12 $$\begin{aligned} p(t,a)= \textrm{expit}\left( f(t,a)\right) = \frac{1}{1 + e^{\left( - f(t,a)\right) }} = \frac{e^{f(t,a)}}{1 + e^{ f(t,a)}}. \end{aligned}$$

The term “expit” refers to the logistic function, defined as $\textrm{expit}(x) = \frac{1}{1 + e^{-x}}$. The name “expit” is used because it represents the inverse of the logit function. By applying the “expit” function to $f(t, a)$, we map the polynomial to the range $(0, 1)$, making it suitable for modeling probabilities, such as prevalence. Thus, the function (12) is a strong candidate for approximating a proper solution to the PDE (1), as supported by the following lemma.

#### Lemma 1

Let

$$\begin{aligned} p(t,a):=\textrm{expit}\left( f(t,a)\right) = \frac{1}{1 + e^{\left( - f(t,a)\right) }} \end{aligned}$$

where $f=f(t,a)$ is a polynomial in *t* (the calendar-time variable) and in *a* (the age variable) with sufficiently high degrees in both variables. Then there exist transition functions $i=i(t,a)$ and $\Delta m = \Delta m(t,a)$ such that (12) is a solution of the PDE (1).

#### Proof

Let $p(t,a)=\textrm{expit}\left( f(t,a)\right)$ and let $\partial := \left( \frac{\partial }{\partial t} + \frac{\partial }{\partial a} \right)$ for abbreviation. Then

13 $$\begin{aligned} \partial p = p\cdot (1-p)\cdot \partial f. \end{aligned}$$

On the other hand, if (12) is a solution of the Transport Eq. (1), it must hold:

14 $$\begin{aligned} \partial p = (1-p)\left( i-p\cdot \Delta m \right) \end{aligned}$$

From (13) and (14) we conclude, that $p(t,a)=\textrm{expit}\left( f(t,a) \right)$ is a solution of (1) if

$$\begin{aligned} p\cdot \partial f = \left( i-p\cdot \Delta m \right) ~~ \Leftrightarrow \end{aligned}$$

15 $$\begin{aligned} i = p\cdot \left( \partial f + \Delta m \right) . \end{aligned}$$

That is, for given *f* and $\Delta m$, there always exists *i*, namely $i = p\cdot \left( \partial f + \Delta m \right)$, so that (12) is a solution of (1).$\square$

Assuming the degree of the polynomial with respect to *a* to be of $deg(f,a)\ge 2$ will allow us to compute the trace analytically for $deg(f,a)\in \left\{ 2, 3 \right\}$ or at least numerically for $deg(f,a)> 3$ as demonstrated in The calculation of the trace section.

## Results

### Application: type 2 diabetes in Germany

For practical application, we use data on type 2 diabetes in the male population of Germany and are particularly interested in obtaining an analytical solution (i.e., an exact solution), for the trace. To achieve this, we need to solve Eq. (18) which can be done analytically by means of the *quadratic equation formula* if for the degree *n* of the polynomial $f=f(t,a)$ with respect to *a* it holds

$$\begin{aligned} \text {deg}\left( f(\cdot , a) \right) = n\in \{ 2,3 \}. \end{aligned}$$

The degree *k* of the polynomial with respect to *t* can be chosen arbitrarily to best suit the problem at hand. As the diabetes data set used for illustration comprises information at $t=2009$ and $t=2015$, we choose a linear polynomial with respect to *t* such that

$$\begin{aligned} \text {deg}\left( f(t, \cdot ) \right) = 1. \end{aligned}$$

Further, we choose $f=f(t,a)$ a polynomial of order 3 with respect to *a*,

$$\begin{aligned} \text {deg}\left( f(\cdot , a) \right) = 3, \end{aligned}$$

which guarantees both a sufficiently good approximation of the data and the analytical computability of the trace (cf. The calculation of the trace section). We therefore define the polynomial as follows:

16 $$\begin{aligned} f(t,a):=\alpha _0 +\alpha _1 a +\alpha _2 a^2 + \alpha _3 a^3 + \beta _0 t +\beta _1 \left( t\cdot a \right) +\beta _2 \left( t\cdot a^2 \right) +\beta _3 \left( t\cdot a^3 \right) . \end{aligned}$$

#### Data set

**Fig. 4:**
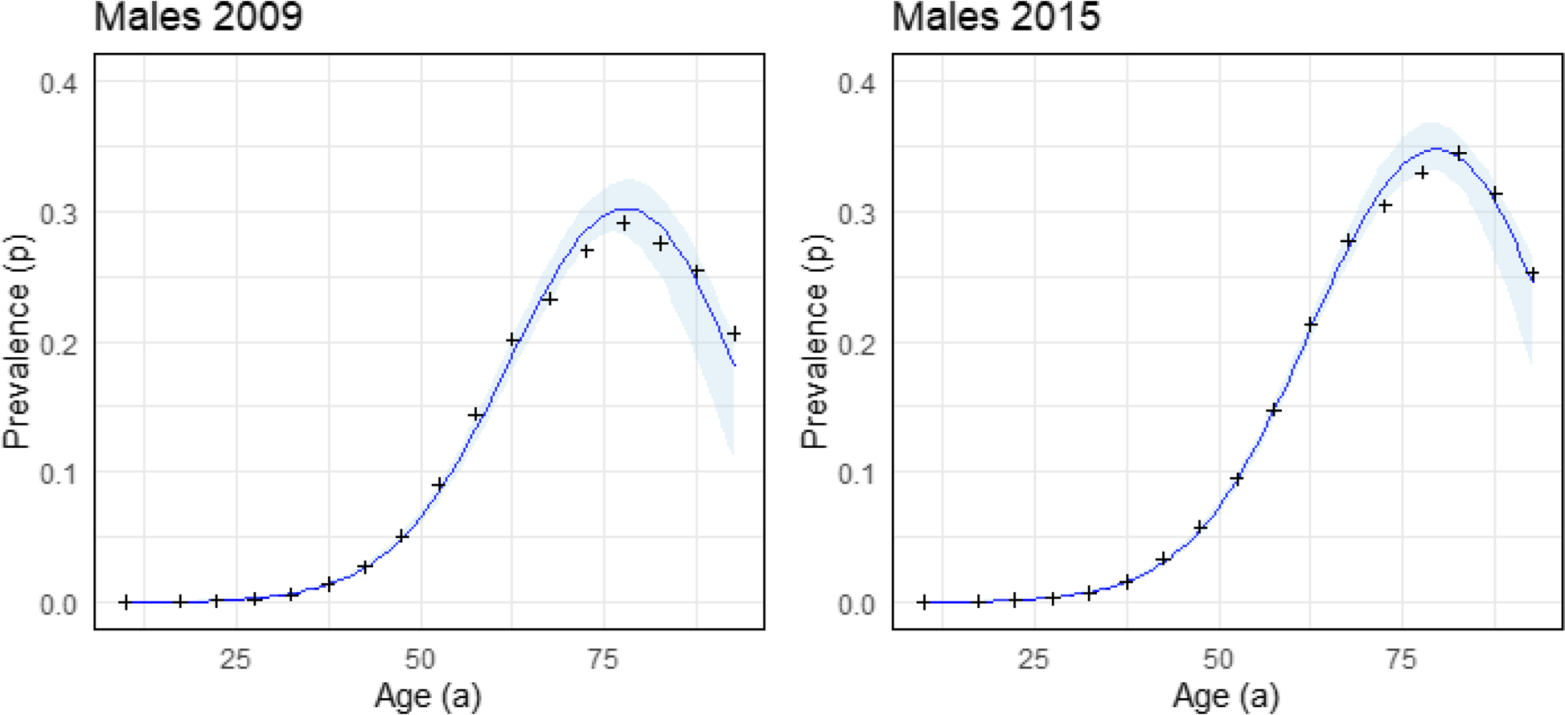
Approximation of type 2 diabetes prevalence using third-order polynomials $f=f(t,a)$ with respect to age *a* (blue line) for $t=2009$ and $t=2015$. Black crosses represent observed data, and light blue areas indicate 95% confidence intervals

As input data set, nationwide billing data of panel physicians that incorporates 85% of the overall German population (approximately 70 million people) is used [6]. This data set includes age- and sex-specific information on the total number of individuals, the number of people with type 2 diabetes, and the corresponding prevalence in the years 2009 and 2015. Figure 4 shows the raw prevalence of type 2 diabetes in men at different ages (+ signs) along with the prevalence approximations derived by applying the expit function (12) to the polynomial (16) (blue lines) in 2009 and 2015. In 2009, the prevalence peaks at approximately 78.4 years of age, while in 2015, it peaks at 78.9. This clearly indicates a prevalence drift between these two years.

#### The calculation of the trace

We performed the approximation of the data with the polynomial (16) and built our solution by means of the logistic function approach (12). Figure 4 demonstrates good agreement between raw data and prevalence fits, represented by blue lines. The 95% bootstrapped confidence intervals (1000 samples), shown in light blue, further support the validity of these approximations.

**Fig. 5:**
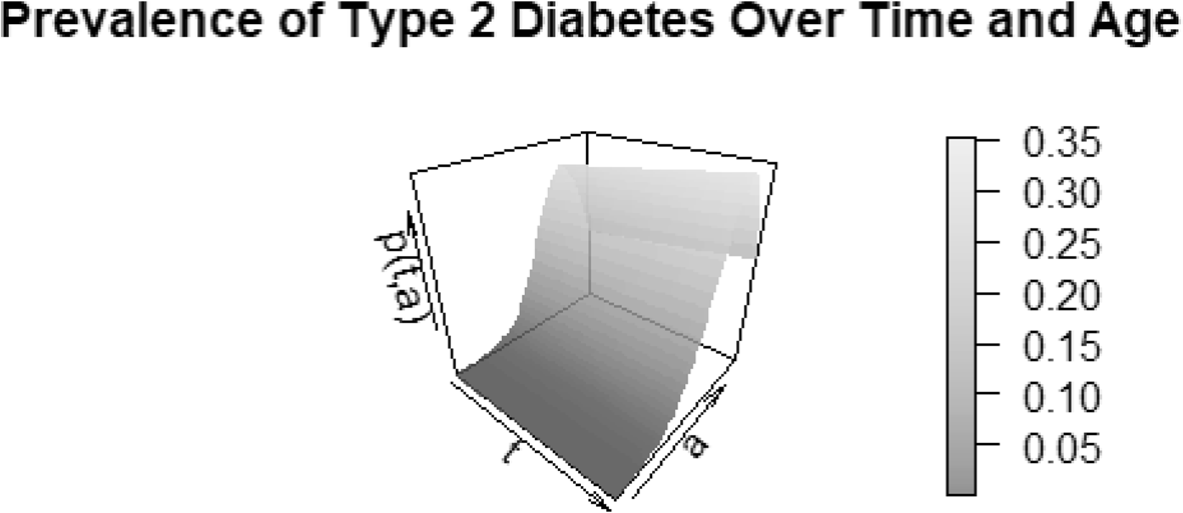
3D plot illustrating the prevalence of type 2 diabetes over time and age. The grayscale gradient on the right represents the prevalence, ranging from 0.05 to 0.35

The evolution of prevalence is most accurately described by Fig. 4 and the prevalence function (12), which combines the two contours into a 3D prevalence function on the Lexis plane, as shown in Fig. 5.

Having an explicit representation of the (approximate) age-dependent prevalence trajectory, we can analytically determine the trace function. For that we observe

17 $$\begin{aligned} \frac{\partial }{\partial a} p(t,a) = p^2(t,a)\cdot \text {exp}\left( -f(t,a) \right) \cdot \frac{\partial }{\partial a}f(t,a). \end{aligned}$$

Since $p^2(t,a)\ge 0$ and $\text {exp}\left( -f(t,a) \right) \ge 0$, from (17) we infer that the necessary condition for the trace, $\frac{\partial }{\partial a} p(t,a)=0$, reduces to

18 $$\begin{aligned} \frac{\partial }{\partial a} f(t,a) = 0. \end{aligned}$$

**Fig. 6:**
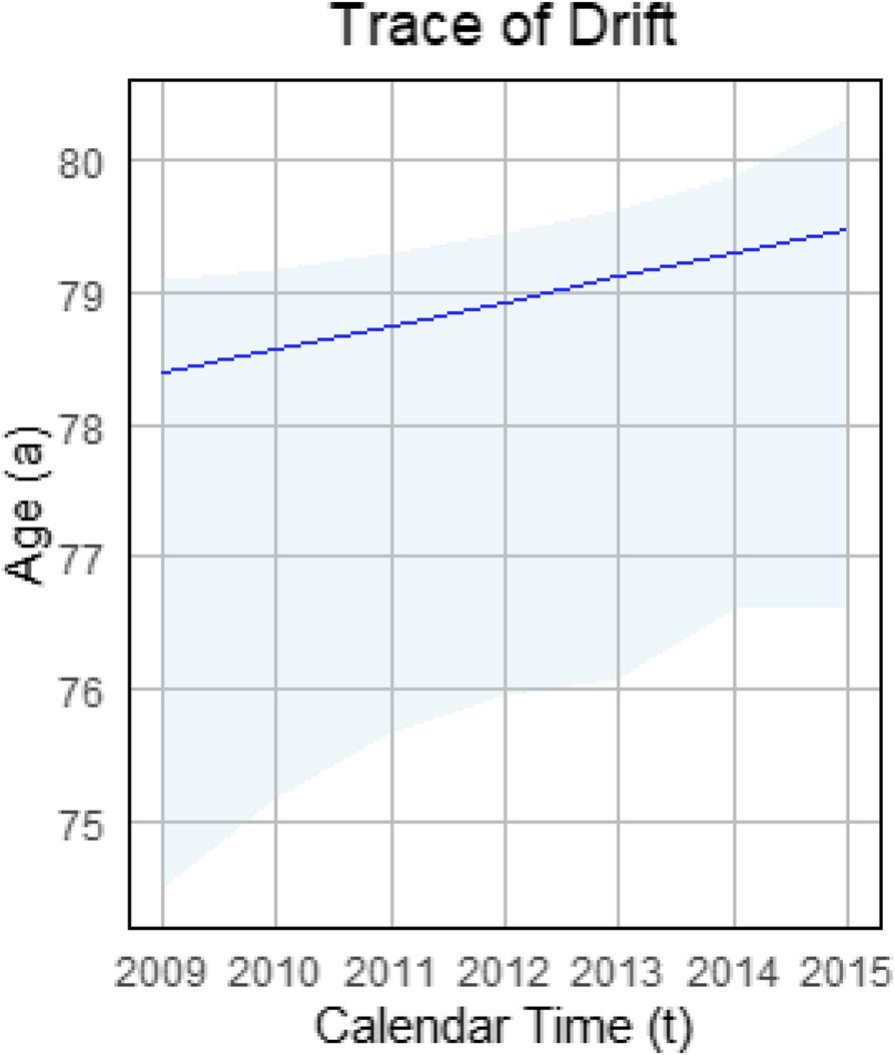
Prevalence peak trace (blue line) on the Lexis plane, with pointwise 95% confidence intervals

Finally, since *f* is a polynomial of degree 3 with respect to *a*, we can solve Eq. (18) to determine the trace function $\hat{a}=\hat{a}(t)$, discarding any non-meaningful solutions, such as negative ages. Figure 6 shows the trace solution including pointwise 95% confidence intervals in light blue at positive ages on the Lexis plane. On the y-axes, the age *a* is given whereas the x-axes indicates calendar-time *t*. The prevalence peak can be seen drifting toward older ages as time progresses, implying that the highest future prevalence of type 2 diabetes will be observed in men over 80 years of age.

#### The correspondence between mortality- and incidence rates

In case of the logistic function approach, a simple calculation shows that the analogous of (9) and (10) read

19 $$\begin{aligned} \frac{\partial }{\partial a}i\left( t, \hat{a}\right) = p\left( t, \hat{a}\right) \cdot \frac{\partial }{\partial a}\Delta m\left( t, \hat{a}\right) + p(t, \hat{a}) \cdot \frac{\partial }{\partial a} \left( \frac{\partial }{\partial t} + \frac{\partial }{\partial a} \right) f\left( t, \hat{a}\right) \end{aligned}$$

and

20 $$\begin{aligned} \text {Pert}\left( t,\hat{a}\right) = \frac{\partial }{\partial a} \left( \frac{\partial }{\partial t} + \frac{\partial }{\partial a} \right) f\left( t, \hat{a}\right) . \end{aligned}$$

It can be shown by differentiation of the polynomial function $f=f(t,a)$ according to (20) and straightforward estimating its magnitude in the Lexis plane, that the Pertubation term (20) has the magnitude of $10^{-3}$ with dimension of variance in the range $10^{-10}$. Therefore, the perturbation from the proportionality (11) can be considered negligible, such that it holds

21 $$\begin{aligned} \frac{\partial }{\partial a}i\left( t, \hat{a}\right) = p\left( t, \hat{a}\right) \cdot \frac{\partial }{\partial a}\Delta m\left( t, \hat{a}\right) \end{aligned}$$

approximately. Thus, on the trace, the age-related change of the incidence $i=i(t,a)$ is approximately proportional to the age-related change in the excess mortality $\Delta m = \Delta m\left( t, \hat{a}\right)$ with proportionality function $p\left( t, \hat{a}\right)$. From a practical point of view this means that the change in incidence is associated with a change in excess mortality, weighted by the prevalence which implicates that knowledge on the prevalence and the temporal age-specific change of the excess mortality is sufficient to estimate the temporal age-specific change in incidence.

With respect to our example data set on type 2 diabetes, we focus for illustration purposes on the age $a_0 = 79$ years (cf. Fig. 6). We calculate the corresponding calendar-time $t_0$ such that $(t_0, a_0)$ belongs to the trace which leads to $t_0 = 2012.41$, rounded to two decimal places. Therefore, the projected change in incidence with respect to age is approximately $p(t_0, a_0)\approx 0.33$ times the change in excess mortality with age:

$$\begin{aligned} \frac{\partial }{\partial a}i\left( t_0, a_0\right) \approx 0.33 \cdot \frac{\partial }{\partial a}\Delta m\left( t_0, a_0\right) , \end{aligned}$$

where $a_0 = 79$ and $t_0 = \hat{a}^{-1}(a_0) \approx 2012.41$. Analogue computations for ages of 70, 80, 90 and 95 years (cf. Table 1) reveal that the proportional factor $p(t,\hat{a})$ tends towards 1, indicating that the age-specific change in incidence is predominantly governed by the age-specific alteration in excess mortality with increasing age.

**Table 1:** Proportionality between change of incidence and change of mortality with respect to age

Age $\varvec{a}_{\varvec{i}}$	Calendar-Time $\varvec{t}_{\varvec{i}} \varvec{=} \hat{\varvec{a}}^{\varvec{-1}}\varvec{(a}_{\varvec{i}}\varvec{)}$	Prevalence $\varvec{p(t}_{\varvec{i}}\varvec{,a}_{\varvec{i}}\varvec{)}$
70	1954	0.07
80	2017	0.37
90	2067	0.83
95	2090	0.94

## Discussion

In this article, we showed that a PDE related to the IDM can be employed to theoretically estimate the temporal change of the peak prevalence of a chronic disease. While the corresponding trace function cannot be analytically computed initially where advanced numerical methods would be required instead, this approach nonetheless leads to the pivotal Eq. (21). This equation facilitates the anticipation of the interrelation between change in incidence and excess mortality with age.

In situations where only aggregated data are available, we employed a fundamental analytical approach to establish the existence of such a trace function which enables us to compute the characteristic trace of the prevalence peak in the Lexis plane. We applied our model to data on type 2 diabetes and found a discernible shift in the prevalence peak over time. This shift can be attributed to modifications in disease progression and mortality rates, as well as alterations in the underlying population demographics.

Our model possesses the capacity to forecast forthcoming patterns in disease prevalence, thereby informing interventions in public health aimed at alleviating the burden posed by chronic diseases.

Specifically, the relationship (21),

$$\begin{aligned} \frac{\partial }{\partial a}i\left( t, \hat{a}\right) \approx p\left( t, \hat{a}\right) \cdot \frac{\partial }{\partial a}\Delta m\left( t, \hat{a}\right) , \end{aligned}$$

holds true in the case of the exemplary application to type 2 diabetes. One important conclusion that can be drawn from this equation is that changes in the age-specific prevalence may have a direct impact on changes of the age-specific incidence. In particular, the equation suggests that if the prevalence increases with age, the incidence may also increase, especially if there is a corresponding increase in excess mortality. Moreover, if excess mortality $\Delta m = \Delta m(t,a)$ can be modelled mathematically or derived from data, similar to the approach taken in [16], the age-specific change in incidence can be directly computed using Eq. (21).

This has important implications for public health policy and planning. For example, if a disease is more prevalent in older people, interventions aimed at reducing the prevalence may have the additional benefit of reducing the incidence among them. Similarly, interventions aiming to reduce excess mortality in older age groups may also have the effect of reducing the age-specific incidence.

Our approach has several advantages. It is mathematically proven and derived, produces plausible results in the case of given aggregated data and its implementation is straightforward. However, it is important to note that Eq. (21) is not valid in general. Determining its validity is challenging due to uncertain and difficult-to-interpret conditions. One possible heuristic approach involves interpreting the error term (20) as an “acceleration” by considering the mixed derivatives of *f* with respect to calendar-time *t* and age *a*. In specific cases, like example (16), the validity of the Eq. (21) can be assessed through direct differentiation of the polynomial function $f=f(t,a)$ and estimating its magnitude. Overall, the equation highlights the importance of understanding the complex interplay between multiple factors in shaping the burden of disease and health outcome in a population, and underscores the need for targeted and multi-faceted interventions to address these issues.

The methods presented in this study serve as a foundational framework for estimating age-specific prevalence drift over time. While our results demonstrate the feasibility of these approaches, a comprehensive investigation into the broader epidemiological implications such as long-term trends, falls beyond the scope of this article. Future studies specifically focusing on these aspects will be essential to further validate the methods and to explore their applicability in public health.

A limitation of Method 2 (the logistic function approach) is that it relies on the availability of reliable data, as evidenced by the uncertainty analysis using a 95% confidence interval in Fig. 4 and 95% pointwise confidence intervals in Fig. 6. While this dependency is not a limitation of our model itself, it underscores the broader challenges associated with data availability.

With Method 2 (the PDE approach), a limitation arises from the absence of a robust numerical scheme for solving the trace ODE in Eq. (7). While standard methods like Runge-Kutta schemes are typically used for such initial value problems, additional refinements are needed due to the complexity of its right-hand side. This complexity arises because the right-hand side of the ODE depends not only on the integral solution of a Riccati-type ODE, but also on the inverse of the age derivative of this solution. Addressing these challenges and developing effective numerical schemes for solving Eq. (7) will therefore be the focus of future work.

In this context, a potential extension of this work could involve deriving a rough linear trend for the drift of the age-dependent prevalence peaks over time by assuming the prevalence peak trajectory to be a linear function. This approach would result in simpler mathematics and improved calculability, albeit at the cost of reduced precision compared to nonlinear predictions. Nevertheless, it could provide a valuable first approximation of the dynamics and represents a promising direction for future research.

## Conclusion

This article introduces two distinct methods for modeling the calendar-time and age-specific prevalence peak drift of chronic diseases.

The first method is a theoretical approach based on a partial differential equation (PDE) derived from the Illness-Death Model (IDM), which links prevalence, incidence, and mortality rates. We derive an ordinary differential equation, Eq. (7), that describes the trajectory of prevalence peaks in the Lexis plane, providing a theoretical framework for tracking how these peaks shift over time and age.

An important result of the PDE approach is the demonstrated relationship between age-specific changes in incidence and excess mortality rates, which are shown to be proportional, with the connection mediated by prevalence, as seen in Eq. (11).

The second method focuses on a logistic function approach that estimates the prevalence drift from aggregated prevalence data. This approach uses polynomial approximation, such as (16), and demonstrates its effectiveness in a practical application. Additionally, 95% confidence intervals have been added to the prevalence approximations and pointwise 95% confidence intervals to the trace of the drift to address the accuracy of the model.

## Supplementary Information

Additional file 1: The Riccati Equation.

Additional file 2: Flow Chart for Method 2.

Additional file 3: How to run the R-code.

Additional file 4: R-Code for reproducing the analysis.

Additional file 5: Dataset for the analysis.

## Data Availability

All data generated or analysed during this study are included in this article and its supplementary information files (“Supplement_data.dat”).

## Abbreviations

IDM: Illness-death model
ODE: Ordinary differential equation
PDE: Partial differential equation
