# Supplementary material for: Modeling the temporal prevalence peak drift of chronic diseases
Source: BMC Med Res Methodol. 2025 Mar 7;25:65. doi: 10.1186/s12874-025-02517-1 (PMC11887115; doi:10.1186/s12874-025-02517-1)
Supplement: Supplementary file 1 — Additional file 1: The Riccati Equation. [file 12874_2025_2517_MOESM1_ESM.pdf]

Appendix A The Ricatti Equation

A.1 On the Solvability of the Ricatti Equation

In this section we will provide a procedure for transforming a *Ricatti*-type ODE in an analytically solvable linear equation if a special solution of the Ricatti ODE is known (for details on this subject see [1]).

For simplicity we let $p' = \frac{\partial}{\partial t}p(t,a)$ and rewrite (5) in the equivalent form

$$p' = (1-p)(i - p\Delta m) \quad \Leftrightarrow \quad p' + (\Delta m + i)p + (-\Delta m)p^2 = i. \tag{A1}$$

Now, for abbreviation let $g := \Delta m + i$ and $h := -\Delta m$ in (A1) to see that (5) indeed is an ODE of *Ricatti*-type:

$$p' + g(t,a)p + h(t,a)p^2 = i(t,a). \tag{A2}$$

In order to transform (A2) into a linear ODE, we will carry out two transformations. First we note that $p_0(t,a) \equiv 1$ is obviously a special solution of (5). Then, we substitute

$$u := p - p_0 = p - 1 \tag{A3}$$

in (A2) to derive at

$$u' + (g(t,a) + 2h(t,a))\, u + h(t,a)u^2 = 0 \tag{A4}$$

which is a *Bernoulli*-type ODE, i.e.

$$u' + k(t)u + l(t)y^{\alpha}$$

with $\alpha = 2$. Finally, substituting

$$z := \frac{1}{u} \tag{A5}$$

into (A4) leads to the linear ODE

$$z' - (g(t,a) + 2h(t,a))\, z = h(t,a), \tag{A6}$$

which can be solved analytically by well known methods. Now, let $z = z(t,a)$ be a solution of (A6). Then the solution of (A2) can easily be calculated by reverse transformation:

$$p(t,a) = u(t,a) + 1 = 1 + \frac{1}{z(t,a)}. \tag{A7}$$

References

[1] Reid, W.T.: Scalar Riccati Differential Equations. In: Riccati Differential Equations, pp. 1–8. Elsevier Science, New York (1972)
