# Supplementary figures and images for: Modeling the temporal prevalence peak drift of chronic diseases
Source: BMC Med Res Methodol. 2025 Mar 7;25:65. doi: 10.1186/s12874-025-02517-1 (PMC11887115; doi:10.1186/s12874-025-02517-1)

## Flowchart for calculating the Trace using Method 2

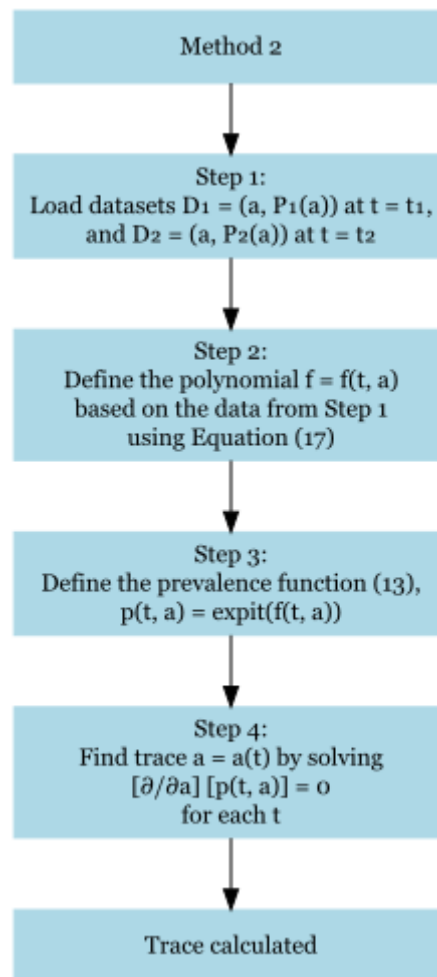

Supplement: Supplementary file 2 — Additional file 2: Flow Chart for Method 2. [file 12874_2025_2517_MOESM2_ESM.pdf]
