# Supplementary material for: Modeling the temporal prevalence peak drift of chronic diseases
Source: BMC Med Res Methodol. 2025 Mar 7;25:65. doi: 10.1186/s12874-025-02517-1 (PMC11887115; doi:10.1186/s12874-025-02517-1)
Supplement: Supplementary file 4 — Additional file 4: R-Code for reproducing the analysis. [file 12874_2025_2517_MOESM4_ESM.pdf]

```
## ############################################################################
## Supplement_R-Code.R:
##
## Supplementary R-Code for Reproducing the Analysis according to the Article
## "Modeling the temporal Prevalence Peak Drift  of Chronic Diseases"
## ############################################################################


## ############################################################################
## RStudio 2024.4.0.735 #######################################################
## R version 4.4.1 (2024-06-14 ucrt) #########################################
## ############################################################################


## ############################################################################
## Load Packages
## ############################################################################
library(plotly)
library(ggplot2)
library(gridExtra)
library(boot)  # Bootstrapping library
rm(list=ls(all=TRUE))

## ############################################################################
## Input Data
## ############################################################################
# Prevalence of type 2 diabetes
Np  <- read.table("N_P.dat", header = TRUE, sep=";")

## ############################################################################
## Model Prevalence Data: Equation (13)
## ############################################################################
logit  <- function(x){log(x/(1-x)) }
expit  <- function(x){1/(1+exp(-x))}

ages <- 15:95
t <- seq(2009, 2015)

ages.p <- c(10, 17.5 + 5*0:15) # class centers of age grps of prv data

pdat   <- data.frame(
  prv = 1e-2 * c(Np$P2009, Np$P2015),
  N = c(Np$N2009, Np$N2015),
  t = rep(c(2009, 2015), each = 34),
  a = rep(ages.p, 4),
  sxM = rep(c(rep(1, 17), rep(0, 17)), 2) # code sex == Male as 1
)

# Fit the polynomial model for males
pmod <- lm(logit(prv) ~ (I(a^3) + I(a^2) + a) * t, data = pdat, subset = (a > 15 & sxM == 1))

# Function to access prevalence
fct_p <- function(time, age){
  pp_ <- expit(predict.lm(pmod, newdata = data.frame(t = rep(time, length(age)), a = age)))
```

```
  return(as.numeric(pp_))
}

## ############################################################################
## Plot Prevalence Curve
## ############################################################################
par(mfrow = c(1, 2), las = 1)
matplot(ages, fct_p(2009, ages), type = "l", lty = 2, col = "blue", ylim = c(0, 0.4),
        main = "Men 2009", xlab = "Age (a)", ylab = "Prevalence (p)")
matplot(ages.p, pdat$prv[1:17], type = "p", pch = 3, add = TRUE)

matplot(ages, fct_p(2015, ages), type = "l", lty = 2, col = "blue", ylim = c(0, 0.4),
        main = "Men 2015", xlab = "Age (a)", ylab = "Prevalence (p)")
matplot(ages.p, pdat$prv[35:51], type = "p", pch = 3, add = TRUE)

## ############################################################################
## Confidence Intervals for Polynomial Approximations
## ############################################################################
# Filter male data
pdat_male <- subset(pdat, sxM == 1)

# Split the male data by year
data_2009 <- subset(pdat_male, t == 2009)
data_2015 <- subset(pdat_male, t == 2015)

# Create prediction dataframes
pred_data_2009 <- data.frame(a = seq(min(pdat_male$a), max(pdat_male$a), length.out = 100))
pred_data_2015 <- data.frame(a = seq(min(pdat_male$a), max(pdat_male$a), length.out = 100))

# Bootstrapping function
boot_fn <- function(data, indices, pred_data) {
  model <- lm(logit(prv) ~ I(a^3) + I(a^2) + a, data = data[indices, ])
  pred_logit <- predict(model, newdata = pred_data)
  return(expit(pred_logit))
}

# Run bootstrapping
set.seed(123)
boot_results_2009 <- boot(data_2009, statistic = function(data, indices) {
  boot_fn(data, indices, pred_data_2009)
}, R = 1000)

boot_results_2015 <- boot(data_2015, statistic = function(data, indices) {
  boot_fn(data, indices, pred_data_2015)
}, R = 1000)

# Calculate confidence intervals
pred_data_2009$fit <- apply(boot_results_2009$t, 2, mean)
pred_data_2009$ymin <- apply(boot_results_2009$t, 2, quantile, probs = 0.025)
pred_data_2009$ymax <- apply(boot_results_2009$t, 2, quantile, probs = 0.975)

pred_data_2015$fit <- apply(boot_results_2015$t, 2, mean)
pred_data_2015$ymin <- apply(boot_results_2015$t, 2, quantile, probs = 0.025)
pred_data_2015$ymax <- apply(boot_results_2015$t, 2, quantile, probs = 0.975)
```

```
## ###############################################################################
## Plot Prevalence Polynomials with Confidence Intervals
## ###############################################################################
plot_2009 <- ggplot() +
  geom_line(data = pred_data_2009, aes(x = a, y = fit), color = "blue", size = 0.5) +
  geom_ribbon(data = pred_data_2009, aes(x = a, ymin = ymin, ymax = ymax), fill = "lightblue",
  alpha = 0.3) +
  geom_point(data = data_2009, aes(x = a, y = prv), color = "black", size = 1, shape = 3) +
  labs(title = "Males 2009", x = "Age (a)", y = "Prevalence (p)") +
  theme_minimal() +
  ylim(0, 0.4)

plot_2015 <- ggplot() +
  geom_line(data = pred_data_2015, aes(x = a, y = fit), color = "blue", size = 0.5) +
  geom_ribbon(data = pred_data_2015, aes(x = a, ymin = ymin, ymax = ymax), fill = "lightblue",
  alpha = 0.3) +
  geom_point(data = data_2015, aes(x = a, y = prv), color = "black", size = 1, shape = 3) +
  labs(title = "Males 2015", x = "Age (a)", y = "Prevalence (p)") +
  theme_minimal() +
  ylim(0, 0.4)

# Arrange plots
combined_plot <- grid.arrange(plot_2009, plot_2015, ncol = 2)
#print(combined_plot)

## ###############################################################################
## Coefficients of p(t,a)
## ###############################################################################
s.dat <- summary(pmod)

# Extract coefficients by name
coeffs <- s.dat$coefficients

# Coefficients of p(t,a) = expit(f(t,a)): Eq. (17)
alpha0 <- coeffs["(Intercept)", "Estimate"]  # Intercept
alpha1 <- coeffs["a", "Estimate"]            # Coefficient of a
alpha2 <- coeffs["I(a^2)", "Estimate"]       # Coefficient of a^2
alpha3 <- coeffs["I(a^3)", "Estimate"]       # Coefficient of a^3

beta0 <- coeffs["t", "Estimate"]             # Coefficient of t
beta1 <- coeffs["a:t", "Estimate"]           # Coefficient of a * t
beta2 <- coeffs["I(a^2):t", "Estimate"]      # Coefficient of a^2 * t
beta3 <- coeffs["I(a^3):t", "Estimate"]      # Coefficient of a^3 * t


## ###############################################################################
## The Prevalence Function p(t,a)
## ###############################################################################
prv <- function(t,a){
  return(expit(alpha0 + alpha1 * a + alpha2 * a^2 + alpha3 * a^3
             + beta0*t + beta1 * a*t + beta2 * a^2*t + beta3 * a^3*t ))
}
```

```
## ############################################################################
## Plot the Trace
## ############################################################################
gamma1 <- function(t) { alpha1 + beta1 * t }
gamma2 <- function(t) { 2 * (alpha2 + beta2 * t) }
gamma3 <- function(t) { 3 * (alpha3 + beta3 * t) }

trace2 <- function(t){
  return(  (-gamma2(t) - sqrt(gamma2(t)^2 - 4 * gamma3(t) * gamma1(t)))/(2 * gamma3(t)) )
}

par(mfrow = c(1, 1))
plot(t, trace2(t), main = "Trace of Drift", xlab = "Calendar Time (t)", ylab = "Age (a)",
type = "l", col = "blue")

## ############################################################################
## Pointwise Confidence Intervals for the Trace
## ############################################################################

bootstrap_trace <- function(data, indices) {
  # Fit the model on the bootstrapped sample
  pmod_boot <- lm(logit(prv) ~ (I(a^3) + I(a^2) + a) * t, data = data[indices, ],
  subset = (a > 15 & sxM == 1))

  # Extract coefficients by name from the bootstrapped model
  coef_boot <- summary(pmod_boot)$coefficients

  # Extract the coefficients using their names from the "Estimate" column
  alpha0 <- coef_boot["(Intercept)", "Estimate"]  # Intercept
  alpha1 <- coef_boot["a", "Estimate"]            # Coefficient of a (linear term)
  alpha2 <- coef_boot["I(a^2)", "Estimate"]       # Coefficient of a^2 (quadratic term)
  alpha3 <- coef_boot["I(a^3)", "Estimate"]       # Coefficient of a^3 (cubic term)

  beta0  <- coef_boot["t", "Estimate"]            # Coefficient of t (linear time term)
  beta1  <- coef_boot["a:t", "Estimate"]          # Coefficient of a * t
  beta2  <- coef_boot["I(a^2):t", "Estimate"]     # Coefficient of a^2 * t
  beta3  <- coef_boot["I(a^3):t", "Estimate"]     # Coefficient of a^3 * t

  # Define gamma functions based on bootstrapped coefficients
  gamma1_boot <- function(t) { alpha1 + beta1 * t }
  gamma2_boot <- function(t) { 2 * (alpha2 + beta2 * t) }
  gamma3_boot <- function(t) { 3 * (alpha3 + beta3 * t) }

  # Calculate the trace using the quadratic formula
  trace_boot <- sapply(t, function(time) {
    (-gamma2_boot(time) - sqrt(gamma2_boot(time)^2 - 4 * gamma3_boot(time) * gamma1_boot(time))) /
    (2 * gamma3_boot(time))
  })

  return(trace_boot)
}


set.seed(123)
```

```
boot_res <- boot(data = pdat, statistic = bootstrap_trace, R = 1000)

reference_trace <- sapply(t, trace2)

lower_ci <- apply(boot_res$t, 2, function(trace_samples) {
  quantile(trace_samples, probs = 0.025)
})

upper_ci <- apply(boot_res$t, 2, function(trace_samples) {
  quantile(trace_samples, probs = 0.975)
})

trace_data <- data.frame(
  Time = t,
  Trace = reference_trace,
  Lower = lower_ci,
  Upper = upper_ci
)


## ###############################################################################
## Plot the Trace with pointwise Confidence Intervals
## ###############################################################################
trace_plot <- ggplot(trace_data, aes(x = Time)) +
  geom_line(aes(y = Trace), color = "blue") +  # Reference trace line
  geom_ribbon(aes(ymin = Lower, ymax = Upper), fill = "lightblue", alpha = 0.2) +
  labs(
    title = "Trace of Drift",
    x = "Calendar Time (t)",
    y = "Age (a)"
  ) +
  theme_minimal() +
  theme(
    plot.title = element_text(hjust = 0.5),
    aspect.ratio = 9/8,
    panel.border = element_rect(colour = "black", fill = NA, size = 0.5),
    panel.grid.minor = element_blank(),
    panel.grid.major.x = element_line(color = "grey", size = 0.5),
    panel.grid.major.y = element_line(color = "grey", size = 0.5)
  ) +
  scale_x_continuous(breaks = seq(2009, 2015, by = 1)) +
  scale_y_continuous(breaks = seq(75, 82, by = 1)) +
  guides(fill = FALSE, color = FALSE)

print(trace_plot)


## ###############################################################################
## Perturbation Term Pert = Pert(t, a)
## ###############################################################################
D_pert <- function(t, ages){
  return(2 * alpha2 + beta1 + (6 * alpha3 + 2 * beta2) * ages + 3 * beta3 * ages^2 +
  (2 * beta2 + 6 * beta3 * ages) * t)
}
```

```
c_time <- seq(from=2009, to=2015, length.out=(2015 - 2009) * 365)
a_time <- trace2(c_time)
D <- D_pert(c_time, a_time)

D_mean <- summary(D)[4]
print(paste("Mean of perturbation from proportionality on the Trace =", D_mean))

D_var <- var(D)
print(paste("Variance of Perturbation on the Trace =", D_var))
```
